# Immature and mature species of the human Prostacyclin Receptor are ubiquitinated and targeted to the 26S proteasomal or lysosomal degradation pathways, respectively

**DOI:** 10.1186/1750-2187-4-7

**Published:** 2009-09-25

**Authors:** Peter D Donnellan, B Therese Kinsella

**Affiliations:** 1 School of Biomolecular and Biomedical Sciences, Conway Institute of Biomolecular and Biomedical Research, University College Dublin, Belfield, Dublin 4, Ireland

## Abstract

**Background:**

The human prostacyclin receptor (hIP) undergoes agonist-induced phosphorylation, desensitisation and internalisation and may be recycled to the plasma membrane or targeted for degradation by, as yet, unknown mechanism(s).

**Results:**

Herein it was sought to investigate the turnover of the hIP under basal conditions and in response to cicaprost stimulation. It was established that the hIP is subject to low-level basal degradation but, following agonist stimulation, degradation is substantially enhanced. Inhibition of the lysosomal pathway prevented basal and agonist-induced degradation of the mature species of the hIP (46-66 kDa). Conversely, inhibition of the proteasomal pathway had no effect on levels of the mature hIP but led to time-dependent accumulation of four newly synthesised immature species (38-44 kDa). It was established that both the mature and immature species of the hIP may be polyubiquitinated and this modification may be required for lysosomal sorting of the mature, internalised receptors and for degradation of the immature receptors by the 26S proteasomes through the ER-associated degradation (ERAD) process, respectively. Moreover, these data substantially advance knowledge of the factors regulating processing and maturation of the hIP, a complex receptor subject to multiple post-translational modifications including N-glycosylation, phosphorylation, isoprenylation, palmitoylation, in addition to polyubiquitination, as determined herein.

**Conclusion:**

These findings indicate that the hIP is post-translationally modified by ubiquitination, which targets the immature species to the 26S proteasomal degradation pathway and the mature species to the lysosomal degradation pathway.

## Background

The prostanoid prostacyclin plays a central role in haemostasis, acting as a potent inhibitor of platelet activation and as a dilator of various types of smooth muscle [1]. It has been implicated as a mediator of inflammation [2] and as an inhibitor of vascular smooth muscle cell (SMC) proliferation *in vitro *[3-5]. Additionally, stable prostacyclin analogs inhibit the proliferation of proximal and distal human pulmonary artery SMCs and human airway SMCs, thereby suggesting their utility in the treatment of such diseases as pulmonary arterial hypertension and asthma [6,7]. Reduced prostacyclin activity, primarily following genetic ablation of the prostacyclin receptor (IP) in mice, has also been linked to increased risk of thrombosis, injury-induced restenosis and atherosclerosis [2,8,9]. Moreover, numerous non-synonymous, in addition to synonymous, mutations have been identified within the coding sequence of the human (h) IP leading to receptor dysfunction which, through genetic linkage studies, have been associated with certain critical vascular disorders including venous thrombosis and intima hyperplasia [10].

The IP, a member of the G-protein-coupled receptor (GPCR) superfamily, primarily couples to activation of Gα_s_/adenylyl cyclase (AC) but may also regulate other secondary effectors in a cell- and species-specific manner including to Gq- activation of phospholipase Cβ [11-16]. Like many other GPCRs, the hIP undergoes *N*-linked glycosylation, a modification important for its membrane localisation, ligand-binding and intracellular signalling [17,18]. Additionally, it is subject to at least two types of post-translational lipid modification, namely palmitoylation at Cys^308 ^and Cys^311 ^[19] and farnesylation/isoprenylation at Cys^383 ^[20] within its proximal and distal carboxyl-terminal (C)-tail domain, respectively. Both palmitoylation and isoprenylation are proposed to result in the formation of a putative double loop structure within its C-tail domain and are required for efficient hIP:G-protein coupling and effector signalling [19,20].

Ligand-induced activation of a given GPCR may typically result in receptor phosphorylation, desensitisation and/or endocytosis leading to internalisation into intracellular compartments or vesicles [21,22]. Internalised receptors may, in turn, then be recycled to the plasma membrane where they are available for another round(s) of signalling or may be targeted for degradation [21,22]. In the case of the hIP, considerable data has been generated regarding the mechanisms responsible for its phosphorylation, internalisation and recycling [17,23-28]. Recent studies have established that Rab5a GTPase plays a critical role in the agonist-induced internalisation of the hIP and that Rab4a and Rab11a are involved in its fast and slow recycling, respectively, to the plasma membrane [24,27]. However, despite these investigations, little is known about the mechanism(s) regulating the turnover or degradation of the hIP under either basal or agonist-induced conditions.

The main systems employed by cells to degrade proteins are the lysosomes and the 26S proteasomes [29]. Lysosomes are vesicular organelles containing a variety of proteases that function in an acidic environment to disrupt peptide bonds [30]. The 26S proteasomes, consisting of a 20S core proteolytic particle and a 19S regulatory particle, generally degrade proteins that have been post-translationally modified by attachment of the polypeptide ubiquitin to the ε-amino group of Lys residue(s) of the substrate in an ATP-dependent manner [31,32]. It is now apparent that a number of GPCRs can be ubiquitinated [33-37]. Agonist-induced ubiquitination of GPCRs does not appear to target them for degradation by the proteasomes, but is instead required for endocytic sorting of the internalised receptors and, ultimately, may target them for lysosomal degradation [38,39]. On the other hand, agonist-independent ubiquitination of a small number of GPCRs has been identified and is mainly associated with targeting of newly synthesised, misfolded GPCRs in the endoplasmic reticulum (ER) for degradation by the cytosolic proteasomes, through a process referred to as ER-associated degradation, or ERAD [40,41].

In this study, it was sought to investigate turnover of the hIP under basal conditions and in response to stimulation with its selective agonist cicaprost. Owing to the relatively low level of endogenous hIP, even within prostacyclin-responsive cells of the vasculature, a previously characterised clonal HEK.hIP cell line which stably over-expresses hemagglutinin (HA)-epitope tagged forms of the hIP was used to facilitate these studies [42]. Our data establish that the hIP is subject to low levels of degradation under basal conditions but that upon agonist stimulation, its degradation is substantially enhanced. Inhibition of the lysosomal pathway prevented basal and agonist-induced degradation of the mature hIP (46-66 kDa). Conversely, while inhibition of the proteasomal degradation pathway had no effect on the level of the mature hIP, it led to a time-dependent accumulation of four newly synthesised immature species of the hIP. Data presented herein also suggest that both the mature and immature species of the hIP may be polyubiquitinated and that this modification may be required for lysosomal sorting of mature, internalised receptors and for degradation of misfolded immature receptors by the 26S proteasomes, respectively.

## Results and Discussion

### Identification of the Species of the hIP Expressed in HEK 293 Cells

**Figure 1:**
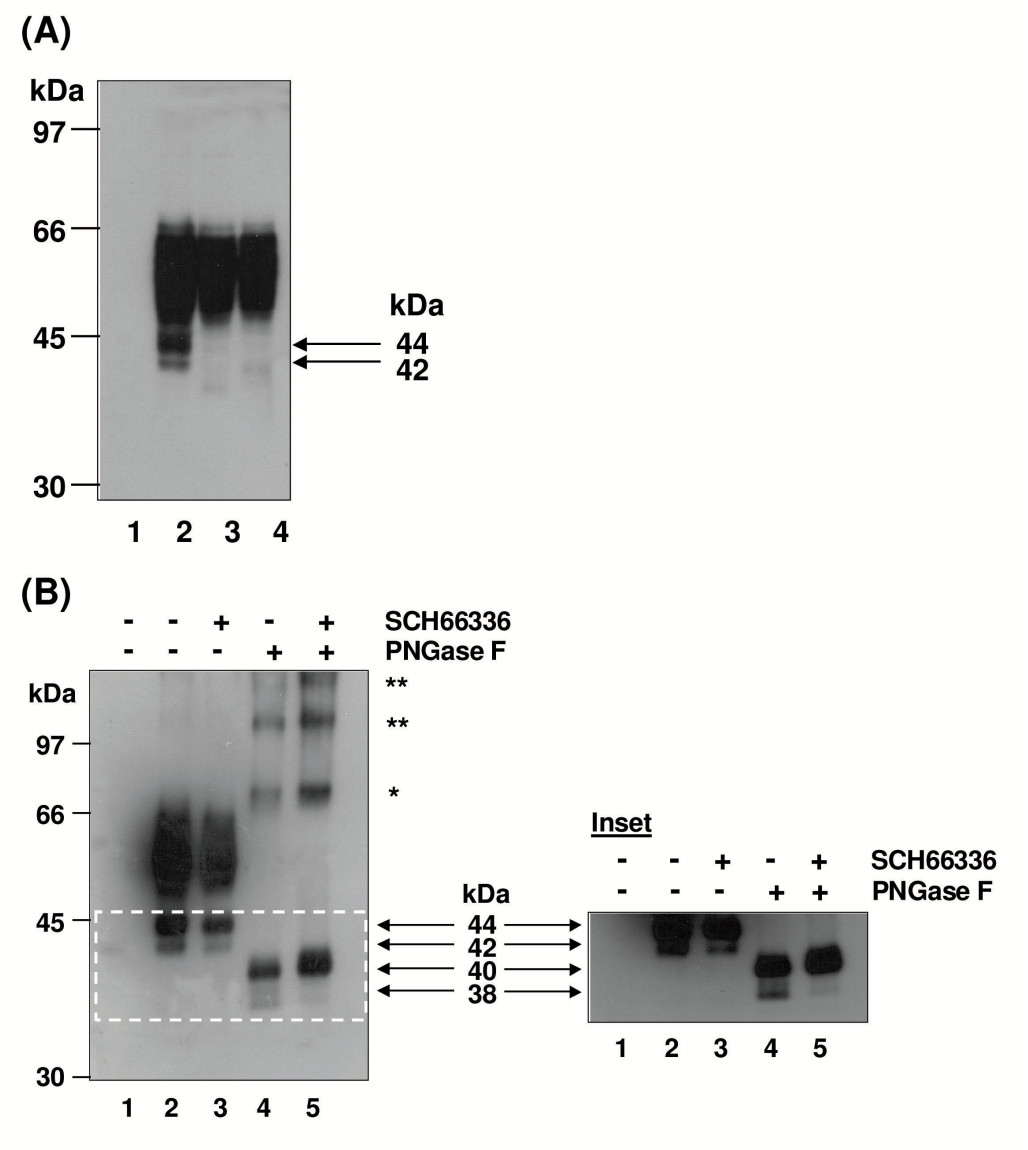
**Identification of the molecular species of the hIP expressed in HEK.hIP cells**. **A**, western blot analysis of whole cell protein (50 μg) from HEK 293 cells (lane 1) or HEK.hIP cells (lanes 2-4) incubated with vehicle (0.001% DMSO, lanes 1, 2 & 4) or tunicamycin (2 μg/ml, lane 3) for 24 hr. Whole cell protein (50 μg) was treated with endoglycosidase H (Endo H, 200 units/50 μg protein; lane 4). **B**, HEK 293 cells (lane 1) or HEK.hIP cells (lanes 2-5) were incubated in the absence (-) or presence (+) of SCH66336 (5 nM) for 24 hr prior to harvesting. Where indicated, whole cell protein (50 μg) was treated with PNGase F (500 units/50 μg protein, lanes 4 & 5). **A **&**B**, reactions were stopped by the addition of SDS-sample Buffer and proteins were resolved by SDS-PAGE, electroblotted onto PVDF membrane and screened with the *anti*-HA (3F10) antibody. **B**, **Inset**, the region highlighted by the dashed-line box in **B **is shown following long-term chemiluminescence exposure. The arrows between the panel and inset highlight the apparent molecular weights (MW) of the core glycosylated, non-farnesylated (44 kDa); core glycosylated, farnesylated (42 kDa); non-glycosylated, non-farnesylated (40 kDa); and non-glycosylated, farnesylated (38 kDa) species of the hIP, respectively. The variably glycosylated mature hIP has an apparent MW of ~46-66 kDa. The higher MW species detected in panel **B**, lanes 4 & 5 may represent dimeric (*) and oligomeric (**) forms of the hIP. The positions of the molecular size markers (kDa) are indicated to the left of the panels. Data are representative of three independent experiments.

The human prostacyclin receptor (hIP) is subject to a number of post-translational modifications, including *N*-linked glycosylation, palmitoylation and farnesylation, amongst others [17-20]. Hence, prior to investigation of turnover of the hIP, it was initially sought to clarify the identity of the molecular species of the hIP expressed in HEK.hIP cells, a clonal mammalian cell line which stably over-expresses hemagglutinin (HA) epitope-tagged forms of the hIP [42]. Consistent with previous reports [17,19], the HA-tagged hIP appeared as a broad, heterogeneous band with an apparent molecular weight (MW) ranging from ~46 - 66 kDa (Figure 1A, lane 2). In addition, a more discrete band was detected at ~44 kDa, with a less abundant species detected at ~42 kDa (Figure 1A, lane 2). None of these proteins were detectable in the host HEK 293 cells (Figure 1A, lane 1).

A variety of reagents were then used to clarify the glycosylation status of the hIP. Following exposure to tunicamycin for 24 hr, the 46 - 66 kDa species of the hIP was still present but the 44 and 42 kDa species of the hIP were not detected (Figure 1A, lane 3). Similarly, treatment of whole cell protein prepared from HEK.hIP cells with endoglycosidase H (Endo H) had no effect on the 46 - 66 kDa species of the hIP, but the 44 and 42 kDa species were not detected (Figure 1A, lane 4). Treatment with peptide N-glycosidase F (PNGase F) led to the disappearance of the 46 - 66 kDa as well as the 44 and 42 kDa species of the hIP and yielded a major species at ~40 kDa and a less abundant form at ~38 kDa (Figure 1B, lane 4; *Inset*: prolonged exposure). Taken together, these data suggest that the broad 46 - 66 kDa immunoreactive band represents the mature species of the hIP containing a variable number of complex *N*-linked glycans, which can only be deglycosylated with PNGase F. On the other hand, the sensitivity of the 44 and 42 kDa bands to tunicamycin and/or Endo H treatment suggests that these may represent newly synthesised, endoplasmic reticulum (ER)-localised species of the hIP that are core glycosylated, while the 40 and 38 kDa forms may represent newly synthesised, non-glycosylated species or intermediates of the hIP that have had their core glycosylation units removed. The inability to detect the 44 and 42 kDa species of the hIP following tunicamycin or Endo H treatment will be addressed at a later point herein.

As stated, the hIP is subject to farnesylation/isoprenylation at Cys^383 ^within an evolutionary conserved -'CAAX' motif [20], an immediate post-translational modification that involves subsequent proteolysis of the -AAX (-S^384^LC^386^) residues and carboxy-methylation to generate a farnesyl-Cys^383^-methyl ester at the C-terminus of the isoprenylated protein. The nett result of such post-translational modifications is to generate a modified hIP that displays faster migration properties, such as on SDS-PAGE, than that of the nascent, non-modified hIP. Thus, to investigate whether any of the hIP species detected may be farnesylated, HEK.hIP cells were preincubated with the farnesyl transferase inhibitor (FTI) SCH66336 [43-45]. Following SCH66336 treatment, the hIP mainly resolved as the broad 46 - 66 kDa and more discrete 44 kDa species with a significant reduction in the intensity of the 42 kDa species (Figure 1B, lane 3; *Inset*: prolonged exposure). These data suggest that the 44 kDa species of the hIP represents a precursor protein that may be modified by farnesylation to generate the 42 kDa species. Furthermore, when HEK.hIP cells were pretreated with SCH66336 prior to deglycosylation with PNGase F, only the major ~40 kDa, but not the minor 38 kDa, species was detected (Figure 1B, lane 5). Therefore, this 40 kDa species may represent the non-glycosylated, non-farnesylated species of the hIP, while the 38 kDa species, detected only in the absence of SCH66336, may represent the non-glycosylated, farnesylated species of the hIP. It was also noticeable that the hIP was detected as higher order oligomeric forms following PNGase F treatment (Figure 1B, lanes 4 & 5). While these oligomers were detected in the presence or absence of SCH66336, there was a slight decrease in the mobility of each species detected in the presence of SCH66336, suggestive of a lack of farnesylation (Figure 1B, compare lanes 4 & 5). As it is only the fully deglycosylated, PNGase F-treated hIP that forms oligomers, they are most likely to be an artifact of the deglycosylation process itself and hence, are unlikely to be of physiologic or biologic relevance in the cellular setting.

### Investigation of Turnover of the hIP

**Figure 2:**
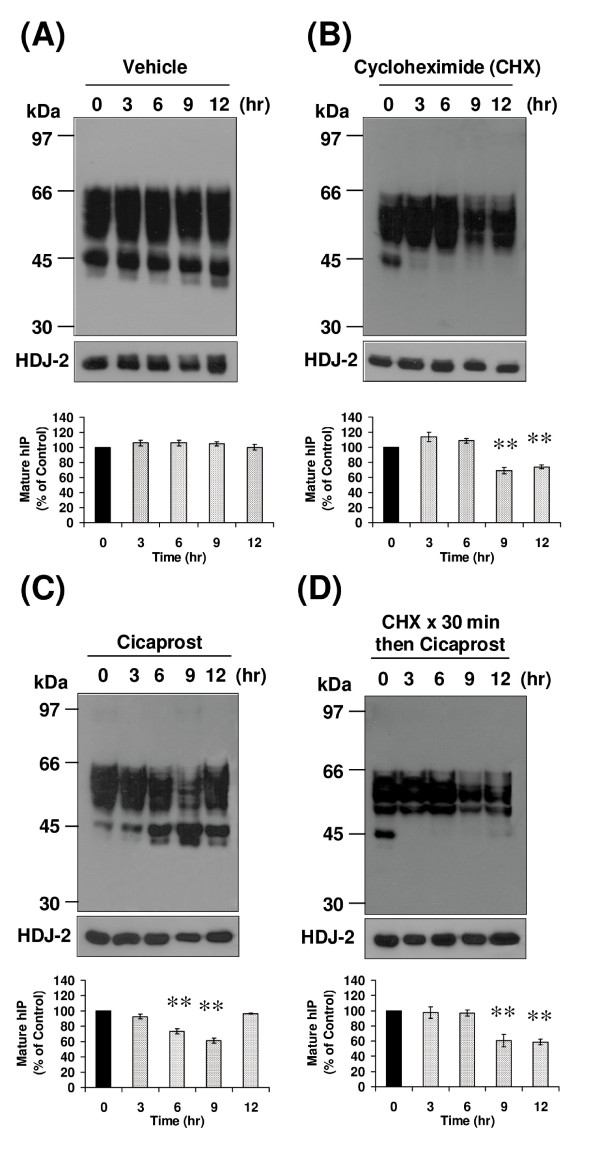
**Investigation of basal and agonist-induced turnover of the hIP**. HEK.hIP cells were incubated with (**A**) vehicle (0.001% DMSO), (**B**) cycloheximide (CHX, 20 μg/ml) or (**C**) cicaprost (1 μM) for 0-12 hr. Additionally, cells were (**D**) pretreated with CHX (20 μg/ml) for 30 min prior to incubation with cicaprost (1 μM) for 0-12 hr. Whole cell protein (50 μg) was resolved by SDS-PAGE, electroblotted onto PVDF membrane and screened with the *anti*-HA (3F10) antibody (**A **- **D**, upper panels). Membranes were stripped and reprobed with the *anti*-HDJ-2 antibody (**A **- **D**, lower panels). The positions of the molecular size markers (kDa) are indicated to the left of the panels. Data are representative of three to six independent experiments. The bar charts show the mean percentage increase or decrease in levels of the mature (46-66 kDa) species of the hIP ± S.E.M. (n = 3-6) where the level of the mature hIP in untreated (0 hr) cells is assigned a value of 100%. **, *p *< 0.01 indicates that the mean percentage levels of the mature hIP was significantly reduced following the respective treatment(s) compared to those levels in untreated (0 hr) cells at that time point.

To examine the turnover of the hIP under basal conditions, HEK.hIP cells were pretreated with cycloheximide (CHX) for 0 - 12 hr to prevent *de novo *receptor synthesis where vehicle (0.001% DMSO)-treated cells served as controls. In all cases, hIP expression levels were analysed by western blot analyses, quantified by densitometry and uniform protein loading was further verified by immunoblotting for HDJ-2, a ubiquitously expressed molecular chaperone protein (Figure 2A - 2D, lower panels). No significant turnover of the 46 - 66 kDa or of the 44 or 42 kDa species of the hIP was apparent over the 12 hr incubation in vehicle-treated cells (Figure 2A). Noteworthy, in general, the 42 kDa species of the hIP was poorly detected at the shorter exposure times, but was clearly evident following prolonged exposures of the immunoblots. In the presence of CHX, the 44 and 42 kDa species of the hIP were not detected 3 hr post-treatment and remained absent upon CHX treatment up to 12 hr (Figure 2B). The level of the 46 - 66 kDa species of the hIP remained stable up to 6 hr following CHX treatment, but from ~9 - 12 hr post-treatment there was a minor but significant reduction in levels of this species compared to vehicle-treated cells (Figure 2B). The CHX did not appear to be effective at inhibiting protein synthesis beyond 9 hr treatment, such that in the presence of CHX the expression of the hIP appears to recover at 9 - 12 hr. Hence, consistent with previous data (Figure 1), these data suggest that the 44 and 42 kDa species represent newly synthesised species of the hIP and, furthermore, that the 46 - 66 kDa species represents the mature hIP, which resolved as a broad band characteristic of its glycosylation pattern in HEK 293 cells [17,18]. Overall, these data suggest that the level of hIP expression is stable under basal conditions, with receptor degradation being compensated for by *de novo *receptor synthesis. However, blocking *de novo *receptor synthesis with the protein synthesis inhibitor CHX revealed minor turnover/degradation of the hIP under basal conditions from ~9 hr post-treatment.

It was next sought to determine the effect of agonist stimulation on the expression and turnover of the hIP. In the presence of cicaprost alone, there was a significant decrease in the level of the mature 46 - 66 kDa species of the hIP, particularly 6 - 9 hr poststimulation, suggesting increased agonist-induced turnover/degradation of the hIP (Figure 2C). However, concurrent with this turnover of the mature hIP, there was also a significant increase in levels of the newly synthesised 44 and 42 kDa species (Figure 2C). Levels of the mature hIP then returned to levels seen in untreated cells 12 hr poststimulation (Figure 2C). These data suggest that treatment of HEK.hIP cells with agonist for up to 9 hr resulted in significant turnover of the mature hIP; however, levels of the mature hIP were restored following 12 hr and more sustained (24 hr, data not shown) agonist stimulation, most likely due to *de novo *protein synthesis. The molecular basis of the increased levels of the newly synthesized hIP in the presence of cicaprost is currently unknown and may reflect alterations at the transcriptional or translational level, in mRNA or protein stability due to increases in cicaprost-induced signalling. In addition, the possibility that cicaprost itself or, more likely, a second-messenger of cicaprost signalling might act as a pharmacologic chaperone also cannot be excluded.

The effect of inhibition of *de novo *protein synthesis prior to stimulation of HEK.hIP cells with agonist was next investigated. When CHX-treated cells were stimulated with cicaprost, the level of the mature hIP was reduced over time, with maximum turnover of the hIP detected ~9 - 12 hr poststimulation (Figure 2D). CHX blocked the synthesis of the 44 and 42 kDa species of the hIP (Figure 2D), thereby preventing replenishment of the levels of the mature hIP from *de novo *synthesis. Overall, these data suggest that minor turnover/degradation of the hIP occurs under basal conditions, but sustained agonist stimulation results in more substantial turnover of the hIP.

### Investigation of Turnover of the hIP in the Presence of Lysosomal Inhibition

**Figure 3:**
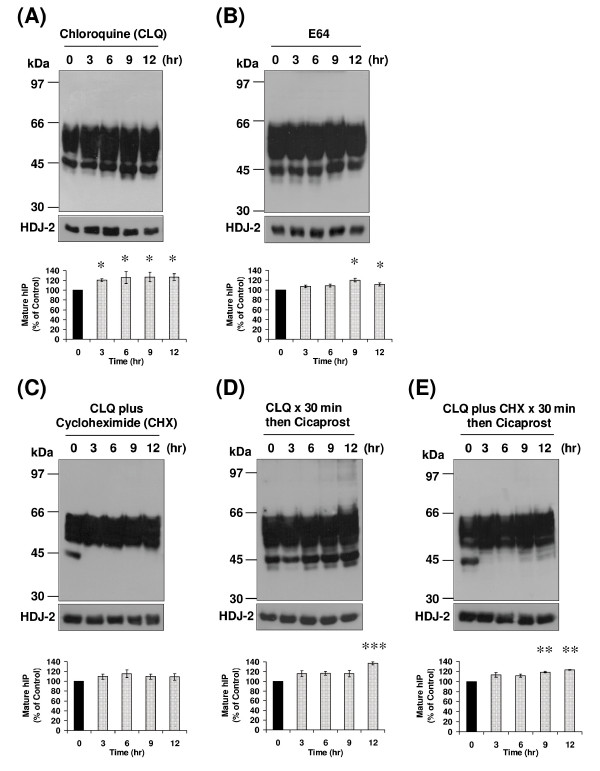
**Effect of lysosomal inhibition on basal and agonist-induced turnover of the hIP**. HEK.hIP cells were incubated with (**A**) chloroquine (CLQ, 100 μM), (**B**) E64 (20 μM) or (**C**) CLQ (100 μM) plus cycloheximide (CHX, 20 μg/ml) for 0 - 12 hr. Alternatively, cells were pretreated with either (**D**) CLQ for 30 min or (**E**) CLQ plus CHX for 30 min prior to incubation with cicaprost (1 μM) for 0-12 hr. **A **- **E**, whole cell protein (50 μg) was resolved by SDS-PAGE, electroblotted onto PVDF membrane and screened with the *anti*-HA (3F10) antibody (upper panels). Membranes were stripped and reprobed with the *anti*-HDJ-2 antibody (**A **- **E**, lower panels). The positions of the molecular size markers (kDa) are indicated to the left of **A **- **E**. Data are representative of three to five independent experiments. The bar charts show the mean percentage increase or decrease in levels of the mature (46-66 kDa) species of the hIP ± S.E.M. (n = 3 - 5) where the level of the mature hIP in untreated (0 hr) cells is assigned a value of 100%. *, *p *< 0.05, **, *p *< 0.01 and ***, *p *< 0.001 indicates that the mean percentage levels of the mature hIP was significantly increased following the respective treatment(s) compared to those levels in untreated (0 hr) cells at that time point.

The majority of cellular proteins are degraded through the lysosomal or the 26S proteasomal systems [29]. Therefore, it was initially sought to investigate the role of lysosomal inhibition on turnover of the hIP. To this end, the effect of treatment of HEK.hIP cells with the lysosomal inhibitors chloroquine (CLQ), a weak base that disrupts the action of lysosomal proteases by altering the pH within the lysosomes [29], and E64, an alternative lysosomal Cys protease inhibitor, was investigated. Levels of the mature, 46 - 66 kDa species and of the 44 and 42 kDa species of the hIP were not reduced but actually were modestly, and significantly, increased following treatment of HEK.hIP cells with CLQ or E64 for 0 - 12 hr (Figure 3A &3B). Basal turnover of the hIP was then investigated in the presence of CLQ plus CHX. The 44 and 42 kDa species of the hIP were not detected 3 hr post-treatment (Figure 3C), further confirming that these species represent newly synthesised receptors. Moreover, no degradation of the mature hIP was evident when cells were treated with CLQ plus CHX for up to 12 hr (Figure 3C), thereby suggesting involvement of the lysosomes in basal degradation of the mature hIP.

Thereafter, the effect of CLQ on agonist-induced turnover of the hIP was examined. Pretreatment of HEK.hIP cells with CLQ prior to stimulation with cicaprost blocked the agonist-induced turnover and actually led to an increase in levels of the mature, 46 - 66 kDa species of the hIP over the duration of the time course (Figure 3D). Levels of the 44 and 42 kDa species also increased significantly over time (Figure 3D), suggesting that cicaprost stimulation increased synthesis of new receptors. Pretreatment of HEK.hIP cells with CLQ plus CHX prior to cicaprost stimulation resulted in the disappearance of the 44 and 42 kDa newly synthesised species of the hIP, while levels of the mature hIP were stable, and in fact significantly increased at the later treatment times (Figure 3E). Overall, while the role of the lysosome on the turnover of the mature hIP appears to be quite low, this is reflective of the fact that its rate of turnover is also low. However, data obtained from studies employing the lysosomal inhibitor CLQ do indeed suggest that both basal- and agonist-induced degradation of the mature, 46 - 66 kDa species of the hIP may occur in lysosomes, as pretreatment of HEK.hIP cells with CLQ in the presence of CHX and/or cicaprost prevented degradation of the 46 - 66 kDa species of the hIP. Such turnover of the mature hIP by the lysosomal degradative pathway may be relatively slow and, therefore, may be required to maintain a balance between *de novo *protein synthesis and degradation.

### Investigation of Turnover of the hIP in the Presence of Proteasomal Inhibition

**Figure 4:**
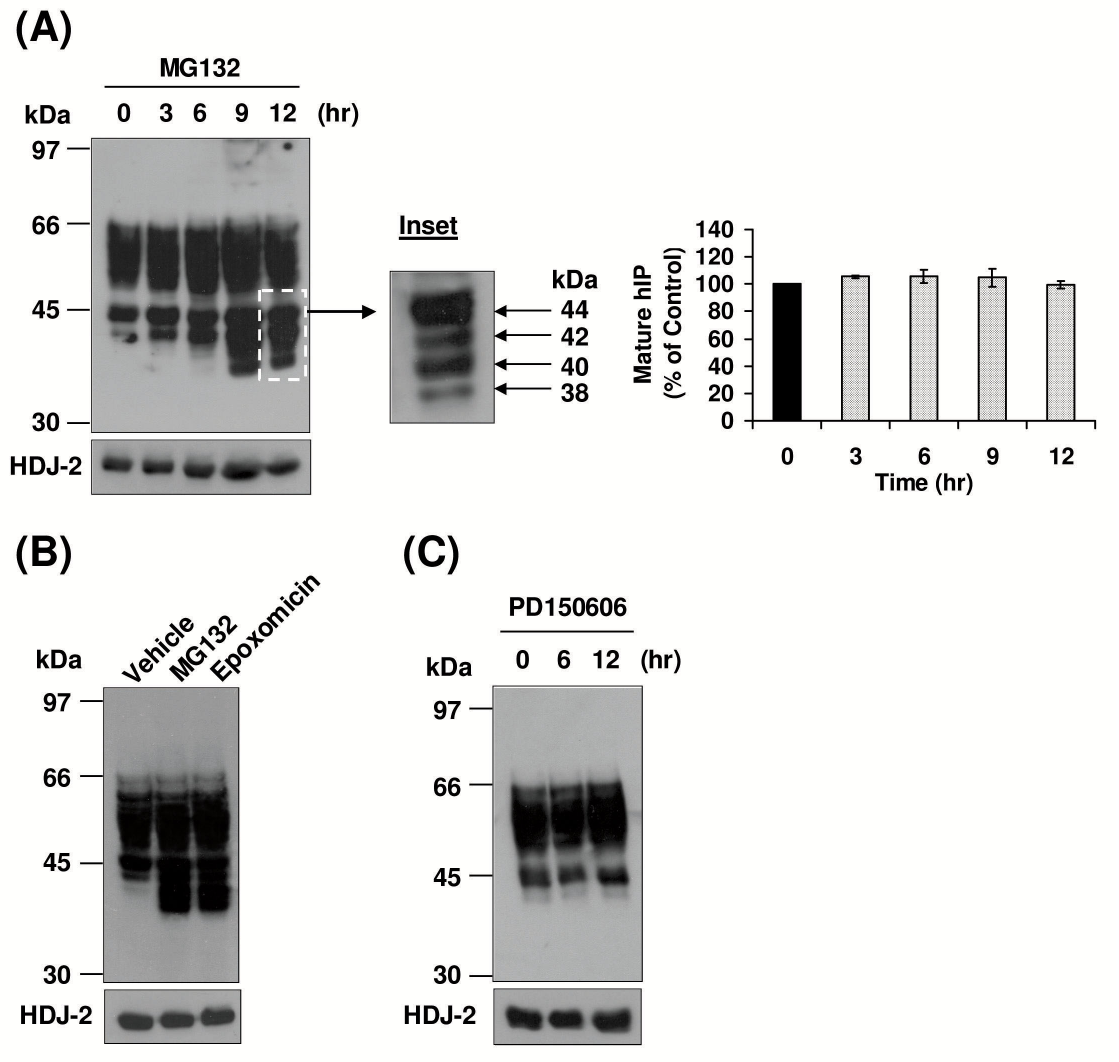
**Effect of proteasomal inhibition on the hIP expressed in HEK.hIP cells**. **A**, HEK.hIP cells were incubated with MG132 (10 μM) for 0-12 hr. Whole cell protein (50 μg) was resolved by SDS-PAGE, electroblotted onto PVDF membrane and screened with the *anti*-HA (3F10) antibody (upper panel). **Inset**, the region highlighted by the dashed-line box in **A **is shown following short-term chemiluminescence exposure where the arrows to the right of the **Inset **indicate the presence of the four predominant bands that accumulate in HEK.hIP cells following proteasomal inhibition with MG132. These bands correspond to the core glycosylated, non-farnesylated (44 kDa); core glycosylated, farnesylated (42 kDa); non-glycosylated, non-farnesylated (40 kDa); and non-glycosylated, farnesylated (38 kDa) species of the hIP, respectively. Alternatively, HEK.hIP cells were incubated with (**B**) MG132 (10 μM) or epoxomicin (0.1 μM) for 12 hr or (**C**) PD150606 (20 μM) for 0, 6 or 12 hr. **B **&**C**, whole cell protein (50 μg) was resolved by SDS-PAGE, electroblotted onto PVDF membrane and screened with the *anti*-HA (3F10) antibody (upper panels). In all cases, membranes were stripped and reprobed with the *anti*-HDJ-2 antibody (**A **- **C**, lower panels). The positions of the molecular size markers (kDa) are indicated to the left of **A **- **C**. Data are representative of three to five independent experiments. The bar chart to the right of **A **shows the mean percentage increase or decrease in levels of the mature (46-66 kDa) species of the hIP ± S.E.M. (n = 5) where the level of the mature hIP in untreated (0 hr) cells is assigned a value of 100%.

It was next sought to investigate the effect of inhibition of the proteasomal degradation pathway on turnover of the hIP. Initially, the selective proteasomal inhibitor MG132, a peptide aldehyde which reversibly inhibits the chymotrypsin-like active site of the proteasomes, was employed [46,47]. Treatment of HEK.hIP cells with MG132 for 0 - 12 hr had no effect on levels of the mature, 46 - 66 kDa species of the hIP (Figure 4A). However, strikingly, proteasomal inhibition led to the rapid and time-dependent accumulation of the 44 and 42 kDa species of the hIP and induced the appearance over time of two further lower MW species of the hIP at ~40 and 38 kDa, respectively (Figure 4A). These low MW species of the hIP that accumulate upon MG132 treatment are shown more clearly in the inset to Figure 4A. At this shorter exposure time, the species that accumulate can be clearly distinguished as four distinct species at ~44, 42, 40 and 38 kDa. The failure to detect accumulation of the mature hIP in the presence of MG132 suggests that the mature hIP may not be degraded by the proteasomes.

MG132 is a highly selective agent that inhibits the activity of the 26S proteasomes. However, it is also reported to inhibit the activity of other cellular proteases, such as lysosomal Cys proteases or calpains [46]. Therefore, to investigate if the effects observed above in HEK.hIP cells using MG132 were specific to the proteasomes, the effect of pretreatment of HEK.hIP cells with other selective proteasome inhibitors was examined. To this end, epoxomicin, a natural epoxyketone which is one of the most selective inhibitors of the proteasome known, and lactacystin, a non-peptide natural product that is structurally different than MG132, were employed [46,48]. Similar to MG132, the four low MW species of the hIP were detected when HEK.hIP cells were treated with epoxomicin (Figure 4B) or lactacystin (data not shown) for 12 hr. Furthermore, pretreatment of HEK.hIP cells with the calpain inhibitor PD150606 had no effect on the pattern of expression of the hIP when compared to vehicle-treated cells (Figure 4C). Combined, these data suggest that the effects attributed to treatment of HEK.hIP cells with MG132, particularly the accumulation of the low MW species of the hIP at 44, 42, 40 and 38 kDa, were specific to its inhibitory effect on the 26S proteasomes and not on other cellular proteases, such as the calpains.

**Figure 5:**
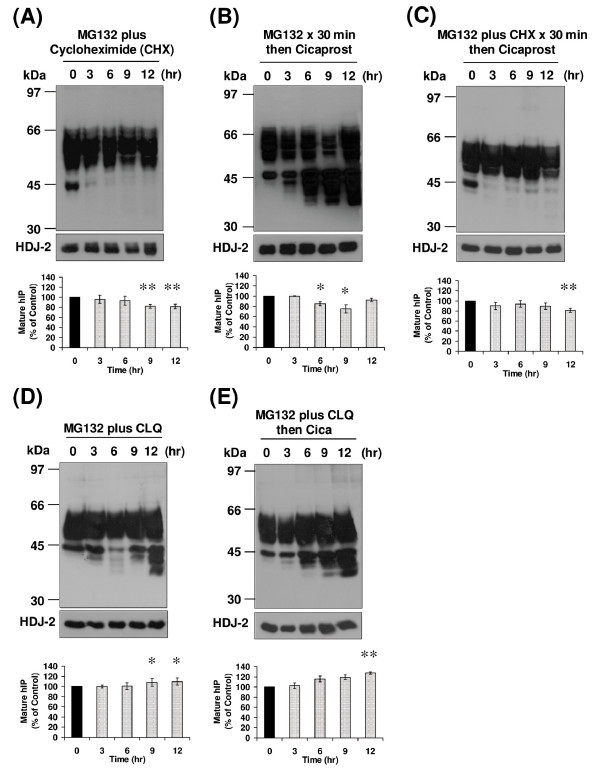
**The effect of proteasomal and lysosomal inhibitors on basal and agonist-induced turnover of the hIP**. HEK.hIP cells were incubated with (**A**) MG132 (10 μM) plus cycloheximide (CHX, 20 μg/ml), (**B**) MG132 (10 μM) for 30 min prior to incubation with cicaprost (1 μM), (**C**) MG132 (10 μM) plus CHX (20 μg/ml) for 30 min prior to incubation with cicaprost (1 μM), (**D**) MG132 (10 μM) plus CLQ (100 μM) or (**E**) MG132 (10 μM) plus CLQ (100 μM) for 30 min prior to incubation with cicaprost (1 μM) for 0-12 hr. **A **- **E**, whole cell protein (50 μg) was resolved by SDS-PAGE, electroblotted onto PVDF membrane and screened with the *anti*-HA (3F10) antibody (upper panels). In all cases, membranes were stripped and reprobed with the *anti*-HDJ-2 antibody (**A **- **E**, lower panels). The positions of the molecular size markers (kDa) are indicated to the left of **A **- **E**. Data are representative of three to five independent experiments. The bar charts show the mean percentage increase or decrease in levels of the mature (46-66 kDa) species of the hIP ± S.E.M. (n = 3-5) where the level of the mature hIP in untreated (0 hr) cells is assigned a value of 100%. *, p < 0.05 and **, p < 0.01 indicates that the mean percentage levels of the mature hIP was significantly increased or decreased following the respective treatment(s) compared to those levels in untreated (0 hr) cells at that time point.

Thereafter, the effect of MG132 on basal and agonist-induced turnover of the hIP was investigated. Treatment of HEK.hIP cells with MG132 in the presence of CHX prevented the expression of the low MW species of the hIP at 44, 42, 40 and 38 kDa within 3 hr (Figure 5A), again confirming that these all represent newly synthesised species. However, inhibition of the proteasomal degradation pathway with MG132 in the presence of CHX did not prevent basal degradation of the mature hIP (Figure 5A) similar to that observed in cells incubated with CHX alone (Figure 2B), thereby suggesting that the 26S proteasomes are not employed in basal degradation of the mature hIP. Down-regulation of the mature hIP at 6 hr and more especially at 9 hr was apparent when HEK.hIP cells were stimulated with cicaprost in the presence of MG132 (Figure 5B), thereby suggesting that proteasomes are not involved in agonist-dependent down-regulation of the mature hIP. However, intense accumulation of the four low MW species of the hIP was seen from 6 hr poststimulation (Figure 5B). The increased rate of accumulation of these species most likely occurred as a result of increased cicaprost-induced *de novo *receptor synthesis. Consistent with this hypothesis, pretreatment of HEK.hIP cells with MG132 plus CHX followed by incubation with cicaprost for 0 - 12 hr prevented the appearance of the four low MW species of the hIP at 44, 42, 40 and 38 kDa within 3 hr (Figure 5C). Furthermore, while significant degradation of the mature hIP was not evident until ~12 hr post-stimulation (Figure 5C), there was no replenishment of levels of the mature hIP due to the prevention of *de novo *receptor synthesis with CHX. Collectively these data suggest that the newly synthesised, low MW species of the hIP were degraded by the 26S proteasomes. On the other hand, degradation of the mature hIP does not appear to be largely dependent on the proteasomes.

The effect of incubation of HEK.hIP cells with MG132 and CLQ simultaneously was then examined. Accumulation of the newly synthesised, low MW species of the hIP at 44, 42, 40 and 38 kDa was detected over time, while no degradation of the mature, 46 - 66 kDa species of the hIP was evident (Figure 5D). Synthesis and accumulation of the low MW species of the hIP was increased when MG132- and CLQ-pretreated HEK.hIP cells were stimulated with cicaprost (Figure 5E). Taken together, these data suggest that the hIP may be subject to two different modes of degradation in HEK.hIP cells. Firstly, the mature, 46 - 66 kDa species of the hIP may be degraded by a pathway employing the CLQ-sensitive lysosomes. Secondly, the newly synthesised, low MW species of the hIP, which accumulate in the presence of MG132 but not in the presence of CLQ, appear to be degraded by the 26S proteasomes.

### Clarification of the Identity of the Newly Synthesised Species of the hIP

**Figure 6:**
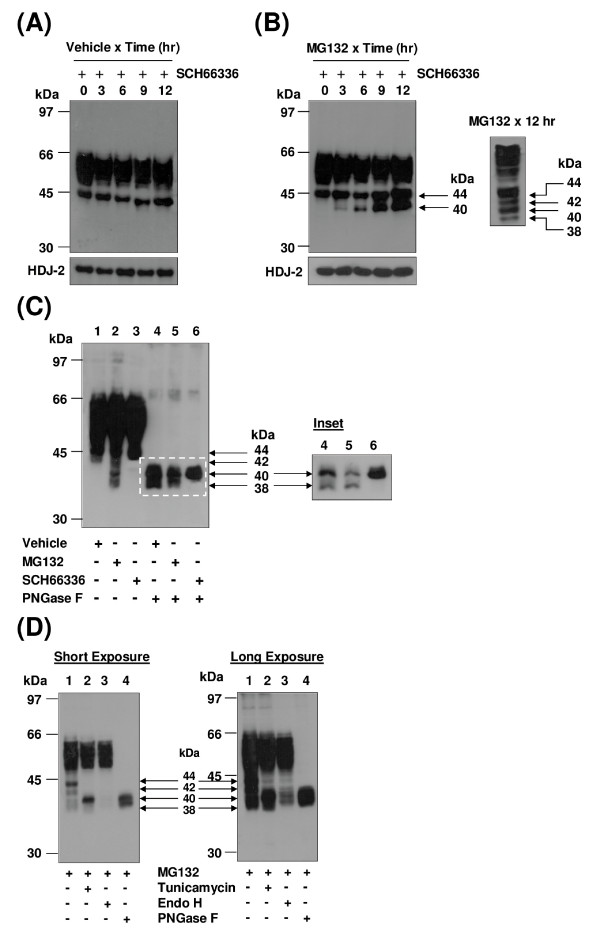
**Clarification of the molecular species of the hIP expressed in HEK.hIP cells**. **A **&**B**, HEK.hIP cells were treated with SCH66336 (5 nM) for 24 hr and then incubated with (**A**) vehicle (0.001% DMSO) or (**B**) MG132 (10 μM) for 0-12 hr prior to harvesting. As a control, HEK.hIP cells were treated with MG132 (10 μM) for 12 hr in the absence of SCH66336 (panel to the right of **B**). Whole cell protein (50 μg) was resolved by SDS-PAGE, electroblotted onto PVDF membrane and screened with the *anti*-HA (3F10) antibody. Membranes were then stripped and reprobed with the *anti*-HDJ-2 antibody (lower panels). **C**, HEK.hIP cells were incubated with vehicle (0.001% DMSO, lanes 1 & 4), MG132 (10 μM, lanes 2 & 5) or SCH66336 (5 nM, lanes 3 & 6) for 12 hr prior to harvesting. Where indicated, whole cell protein (50 μg) was treated with PNGase F (500 units/50 μg protein; lanes 4-6). **Inset**, the region highlighted by the dashed line box in **C **is shown following short-term chemiluminescence exposure. **D**, HEK.hIP cells were pretreated with MG132 (10 μM, lanes 1, 3 & 4) or 10 μM MG132 plus 2 μg/ml tunicamycin (lane 2) for 12 hr prior to harvesting. Thereafter, where indicated, whole cell protein (50 μg) was treated with endoglycosidase H (endo H, 200 units/50 μg protein; lane 3) or PNGase F (500 units/50 μg protein, lane 4), where both short- and long-term exposures are shown. Panels **B - D**, the respective arrows indicate the presence or absence of the core glycosylated, non-farnesylated (44 kDa); core glycosylated, farnesylated (42 kDa); non-glycosylated, non-farnesylated (40 kDa); and non-glycosylated, farnesylated (38 kDa) species of the hIP detected following the various treatments. The positions of the molecular size markers (kDa) are indicated to the left of the panels. Data are representative of three independent experiments.

To further investigate the nature of the species that accumulate in the presence of MG132 and, specifically, to further identify the farnesylated versus non-farnesylated species of the hIP, HEK.hIP cells were preincubated with the FTI SCH66336 for 24 hr prior to treatment with MG132 or the vehicle (0.001% DMSO) for a further 0 - 12 hr. Analysis of total cellular protein from vehicle-treated cells in the presence of SCH66336 indicated that the HA-tagged hIP resolved as a broad 46 - 66 kDa band and as a discrete 44 kDa band (Figure 6A), with no evidence found for the 42 kDa species, even following prolonged exposure to SCH66336 (data not shown). In the presence of MG132 and SCH66336, there was a time-dependent accumulation of the 44 and 40 kDa species of the hIP, but the 42 or 38 kDa species were not detected (Figure 6B). The lane on the right of Figure 6B illustrates the accumulation of all four immature species of the hIP in HEK.hIP cells following treatment with MG132 (12 hr) in the absence of SCH66336. Hence, inhibition of farnesylation prevented the accumulation of the 42 and 38 kDa species of the hIP upon proteasomal inhibition, thereby suggesting that these latter species can indeed be modified by the 15-carbon farnesyl isoprenoid. Additionally, these data suggest that the hIP can be glycosylated without being farnesylated.

It was next sought to clarify whether any of the low MW species at 44, 42, 40 and 38 kDa that accumulate in the presence of MG132 actually correspond to the species of the hIP that are detected in the presence of SCH66336 and/or following PNGase F treatment. Pretreatment of HEK.hIP cells with MG132 followed by deglycosylation with PNGase F resulted in detection of the same two species at 40 and 38 kDa as seen when cells were treated with PNGase F alone (Figure 6C, compare lanes 4 & 5). Pretreatment of HEK.hIP cells with SCH66336 prior to deglycosylation resulted in detection of the 40 kDa species only (Figure 6C, lane 6), which corresponds to the upper species detected when cells were treated with PNGase F alone or in combination with MG132 (Figure 6C, compare lanes 4, 5 & 6). The region indicated by the white dashed-line box in Figure 6C is also shown following short-term chemiluminescence exposure in order to more clearly highlight the presence or absence of these low MW species following the various treatments (Figure 6C, *Inset*).

Treatment of HEK.hIP cells with tunicamycin or Endo H resulted in loss of the 44 and 42 kDa species of the hIP (see Figure 1A). To investigate whether this inability to detect these species was due to their rapid degradation by the 26S proteasomes, HEK.hIP cells were treated with MG132 plus tunicamycin or with MG132 alone prior to deglycosylation of total cellular protein with Endo H. When tunicamycin-treated cells were incubated with MG132, the 44 and 42 kDa species were not detected but the 40 kDa and, to a lesser extent, the 38 kDa species was detected (Figure 6D, lane 2). When total cell protein from MG132-treated HEK.hIP cells was digested with Endo H, the 44 and 42 kDa species were not detected but the 40 and 38 kDa species were detected, especially upon prolonged exposure (Figure 6D, lane 3). Therefore, these data suggest that the tunicamycin- and Endo H-sensitive 44 and 42 kDa species of the hIP may normally be degraded by the 26S proteasomes, thereby accounting for the inability to detect these species in Figure 1A (lanes 3 & 4). Furthermore, these two lower MW species of the hIP migrating at 40 and 38 kDa were similar to the two species detected when HEK.hIP cells were deglycosylated with PNGase F following MG132 treatment (Figure 6D, compare lanes 3 & 4). Combined, these data clarify that the four species that accumulate when HEK.hIP cells are incubated in the presence of the proteasomal inhibitor MG132 correspond to the core glycosylated, non-farnesylated (44 kDa); core glycosylated, farnesylated (42 kDa); non-glycosylated, non-farnesylated (40 kDa); and non-glycosylated, farnesylated (38 kDa) species of the hIP, respectively.

### Investigation of the Subcellular Localisation of the Species of the hIP

**Figure 7:**
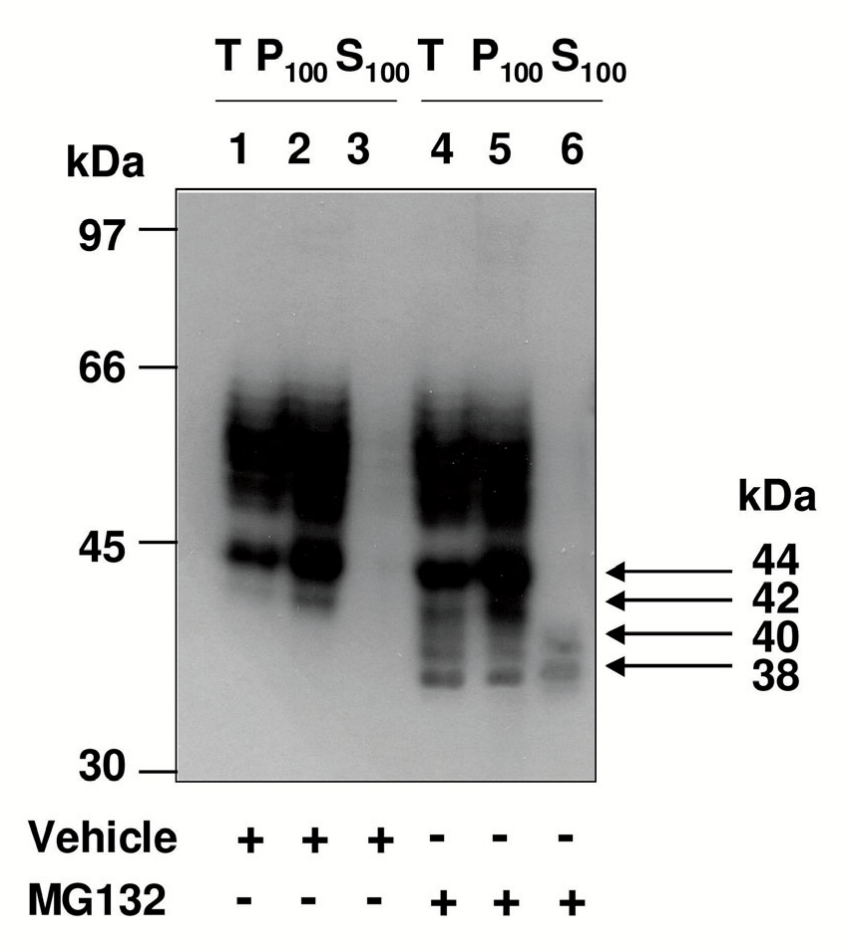
**Subcellular localisation of the hIP in the presence of MG132**. HEK.hIP cells were pretreated in the presence (+) or absence (-) of vehicle (0.001% DMSO, lanes 1-3) or MG132 (10 μM, lanes 4-6) for 12 hr. Aliquots of total (T; 50 μg), membrane (P_100_; 37.5 μg) and soluble (S_100_; 25 μg) fractions were resolved by SDS-PAGE, electroblotted onto PVDF membrane and screened with the *anti*-HA (3F10) antibody. The arrows at the right of the panel indicate the core glycosylated, non-farnesylated (44 kDa); core glycosylated, farnesylated (42 kDa); non-glycosylated, non-farnesylated (40 kDa); and non-glycosylated, farnesylated (38 kDa) species of the hIP, respectively, that accumulate following the various treatments. The positions of the molecular size markers (kDa) are indicated to the left of the panel. Data are representative of three independent experiments.

Thereafter it was sought to examine the subcellular localisation of the various species of the hIP following treatment of HEK.hIP cells with MG132 for 12 hr, where vehicle-treated cells served as controls. The HA-tagged hIP resolved as three major bands at 46 - 66, 44 and 42 kDa, respectively, which were detected in the total (T) and pellet (P_100_, representing crude membrane) fractions of vehicle-treated cells (Figure 7, lanes 1 & 2). In the presence of MG132, the 46 - 66 kDa species and the 44, 42, 40 and 38 kDa species were detected in the T and P_100 _fractions (Figure 7, lanes 4 & 5). However, trace levels of the 40 and 38 kDa receptor species were also present in the soluble, supernatant/S_100 _fraction of MG132-treated (Figure 7, lane 6) but not in the corresponding S_100 _fraction from vehicle-treated cells (Figure 7, lane 3), even following prolonged exposure (data not shown). These data suggest that these latter species represent newly synthesised, non-glycosylated forms of the hIP or intermediates of the hIP that have been deglycosylated and, in both cases, these species may then be targeted for degradation by the cytosolic proteasomes, presumably following their retrotranslocation from the ER in a process known as ER-associated degradation (ERAD) [40,49].

### Effect of Proteasomal Inhibition on Expression and Intracellular Signalling by the hIP

**Figure 8:**
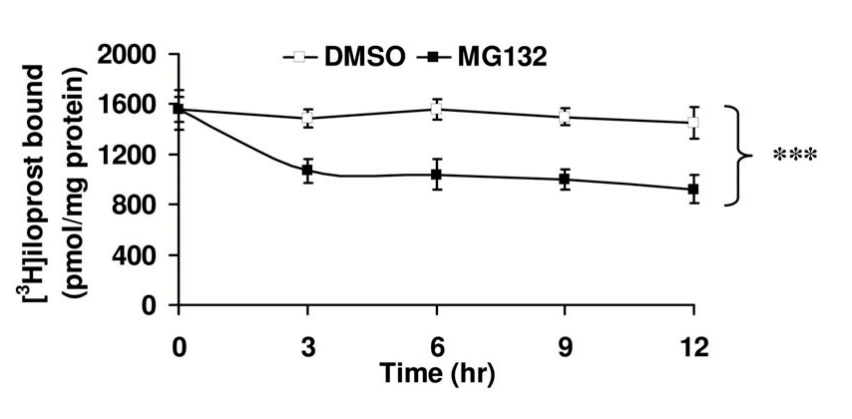
**Effect of MG132 on radioligand binding by the hIP**. HEK.hIP cells were pretreated with DMSO (0.001%) or MG132 (10 μM) for 0-12 hr, with untreated cells serving as the reference. Cells were harvested and radioligand-binding assays were then performed on crude membrane (P_100_) fractions in the presence of 4 nM [^3^H]iloprost at 30°C, as outlined in *Experimental Procedures*. The data are presented as the mean [^3^H]iloprost bound (pmol/mg protein ± S.E.M., n = 4). For untreated cells (0 hr), the actual value for mean [^3^H]iloprost bound (pmol/mg protein ± S.E.M.) was 1.55 ± 0.10 pmol/mg protein. ***, *p *< 0.0001 indicates that the mean [^3^H]iloprost bound (pmol/mg protein) ± S.E.M. was significantly reduced in the presence of MG132 compared to DMSO at each time point.

In view of the significant effects of MG132 on the expression of the hIP, its effect on the level of functional hIP expression as monitored by assessment of radioligand-binding and agonist-induced intracellular signalling of the hIP was then examined. To begin with, the hIP was typically expressed at 1.55 - 1.60 pmol/mg membrane protein in HEK.hIP cells (Figure 8). Incubation with MG132 for 3 - 12 hr significantly reduced expression of the hIP relative to that of control, vehicle (0.001% DMSO)-treated cells (Figure 8, *p *< 0.0001, ANOVA). It was notable that cicaprost alone led to significant reductions in radioligand binding throughout the 0 - 12 hr period (*p *< 0.0001, ANOVA; e.g. at 9 hr, 0.56 pmol/mg membrane protein in HEK.hIP cells). The substantial apparent loss in radioligand binding by the hIP, as opposed to actual level of receptor protein expression, is most likely due to the fact that the hIP is desensitized in the presence of its own agonist cicaprost. Hence, in the presence of cicaprost, specific radioligand binding may not truly reflect actual protein expression.

**Figure 9:**
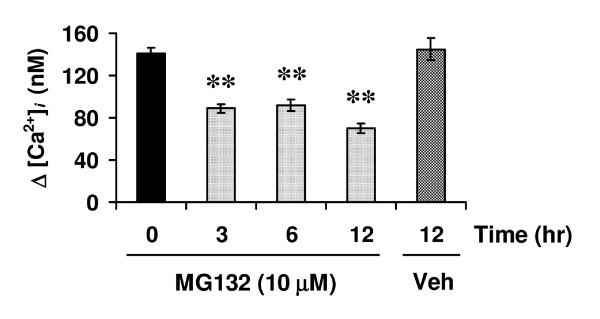
**Effect of MG132 on intracellular calcium ([Ca^2+^]_*i*_) mobilisation by the hIP**. HEK.hIP cells were pretreated with MG132 (10 μM) for 3 hr, 6 hr or 12 hr prior to harvesting, where untreated (0 hr) or vehicle-treated (0.001% DMSO, 12 hr) cells served as controls. Cells were preloaded with Fura2/AM and stimulated with 1 μM cicaprost. In all cases, mean maximal changes in intracellular calcium mobilised (Δ[Ca^2+^]_*i *_(nM) ± S.E.M.) were determined from at least four independent experiments. Actual mean Δ[Ca^2+^]_*i *_± S.E.M. were: 0 hr, 141.2 ± 5.76 nM; vehicle, 12 hr, 145.0 ± 10.3 nM; MG132, 3 hr, 89.0 ± 4.11 nM; MG132, 6 hr, 91.8 ± 5.8 nM; and MG132, 12 hr, 70.4 ± 4.48 nM. Typically, actual baseline [Ca^2+^] levels in HEK.hIP cells were 10-15 nM. In all cases, mean maximal changes in intracellular calcium mobilised (Δ[Ca^2+^]_*i *_(nM) ± S.E.M.) were determined from at least four independent experiments. **, *p *< 0.01 indicates that the mean Δ[Ca^2+^]_*i *_was significantly reduced in the presence of MG132 compared to those levels in untreated cells at the specified time points (3, 6 & 12 hr).

Whilst the hIP primarily couples to Gα_s_/adenylyl cyclase (AC) activation, it may also readily couple to Gα_q_/phospholipase (PL) Cβ activation, leading to mobilisation of Ca^2+ ^from intracellular stores [14]. For convenience, the effect of MG132 on agonist-induced [Ca^2+^]_*i *_mobilisation was examined herein. Stimulation of HEK.hIP cells with cicaprost led to a significant transient rise in [Ca^2+^]_*i *_mobilisation (141.2 ± 5.76 nM, Figure 9; 0 hr). Pretreatment of HEK.hIP cells with MG132 for 3 hr resulted in a 37% reduction in levels of [Ca^2+^]_*i *_mobilised compared to untreated cells (89.0 ± 4.11 nM, Figure 9; *p *< 0.01). Prolonged treatment of HEK.hIP cells with MG132 for 6 or 12 hr decreased levels of [Ca^2+^]_*i *_mobilisation by 35% and 50.2%, respectively, compared to untreated cells (91.8 ± 11.8 nM & 70.4 ± 4.48 nM, respectively, Figure 9; *p *< 0.01). As controls, pretreatment of these cells with vehicle (0.001% DMSO) for up to 12 hr had no significant effect on levels of [Ca^2+^]_*i *_mobilised compared to untreated cells (145.0 ± 10.3 nM, Figure 9 while, in the absence of pre-incubation, MG132 alone had no effect whatsoever on cicaprost-induced [Ca^2+^]_*i *_mobilisation (154.67 nM ± 9.35 nM). Moreover, MG132 had no significant effect on ligand binding or on agonist-induced [Ca^2+^]_*i *_mobilisation by the TPα isoform of the human thromboxane A_2 _receptor, even following prolonged treatment (H. Reid, unpublished data). Collectively, these data suggest that proteasomal inhibition of HEK.hIP cells with MG132 specifically impairs the expression of functional hIP, as observed by significant reductions in both radioligand binding in crude membrane fractions and in agonist-induced [Ca^2+^]_*i *_mobilisation. Moreover, collectively, these data suggest that for efficient expression of functional IP, an intact proteasomal system is required.

### Investigation of Ubiquitination of the hIP

Proteins that are degraded by the 26S proteasomes are typically modified by attachment of ubiquitin moiety(ies) prior to degradation [32]. Furthermore, many GPCRs may be targeted for degradation by the lysosomes upon prior ubiquitination of the internalised receptor [50,51]. Data presented herein suggested that inhibition of the proteasomal degradation pathway in HEK.hIP cells with MG132 prevented the degradation of the newly synthesised species of the hIP, while degradation of the mature, 46 - 66 kDa species of the hIP was prevented following inhibition of the lysosomes. However, these assays were based solely on western blot detection of the hIP and were not analysed for the detection of ubiquitination *per se*. Hence, data generated thus far cannot exclude the possibility that the hIP (immature and/or mature forms) may actually be ubiquitinated. Therefore, it was next sought to investigate whether ubiquitin-modified species of the hIP may be detected in the absence or presence of proteasomal inhibition.

**Figure 10:**
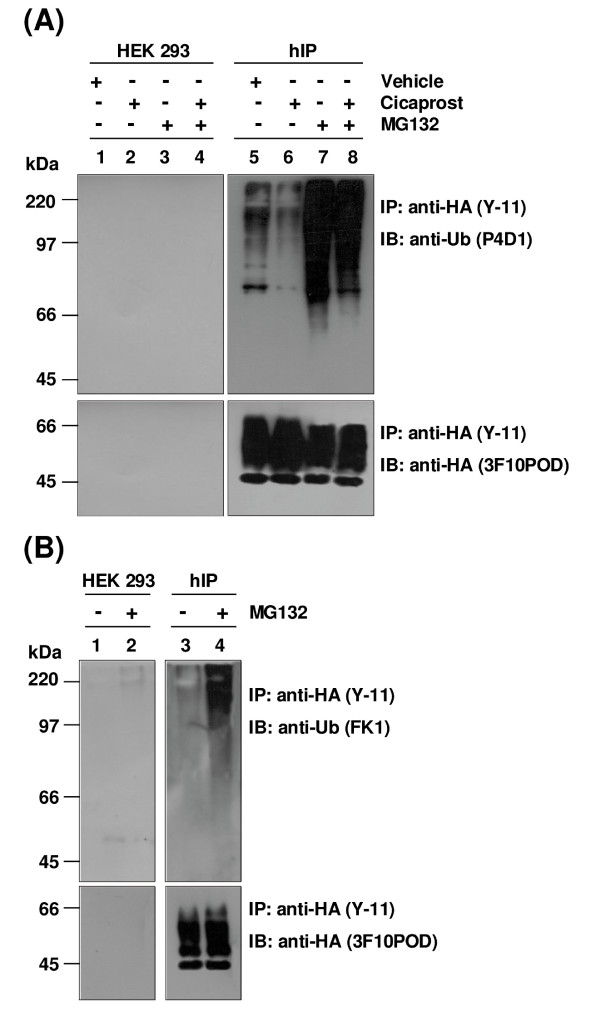
**Immunodetection of ubiquitination of the hIP**. **A**, HEK 293 (lanes 1-4) and HEK.hIP (hIP, lanes 5-8) cells were incubated in the presence (+) or absence (-) of vehicle (0.001% DMSO), cicaprost (1 μM) or MG132 (10 μM) for 4 hr. Additionally, cells were pretreated with MG132 (10 μM) for 30 min prior to incubation with cicaprost (1 μM) for 4 hr. **B**, HEK 293 (lanes 1 & 2) and HEK.hIP (hIP, lanes 3 & 4) cells were incubated in the presence (+) or absence (-) of MG132 (10 μM) for 4 hr. **A **&**B**, thereafter, cells were harvested, lysed in Radio-immunoprecipitation (RIP) Buffer and HA-tagged hIPs were immunoprecipitated (IP) using the *anti*-HA antibody (IP: anti-HA (Y-11)), as described under *Experimental Procedures*. Immunoprecipitates were resolved by SDS-PAGE, followed by electroblotting to PVDF membrane. In each case, membranes were initially immunoblotted (IB) with the *anti*-HA peroxidase-conjugated antibody (IB: *anti*-HA (3F10POD), lower panels) to detect the HA-tagged hIP. **A**, membranes were rescreened with the *anti*-ubiquitin monoclonal antibody (IB: *anti*-Ub (P4D1), upper panel), which recognises both mono- and polyubiquitinated proteins. **B**, membranes were rescreened with the *anti*-ubiquitin monoclonal antibody (IB: *anti*-Ub (FK1), upper panel), which recognises polyubiquitinated proteins only. The positions of the molecular size markers (kDa) are indicated to the left of **A **&**B**. Data are representative of three independent experiments.

To this end, an *anti*-ubiquitin (P4D1) antibody, which recognises both mono- and polyubiquitinated proteins, was employed to specifically screen for ubiquitinated species of the hIP following immunoprecipitation of the HA-tagged hIP with an *anti*-HA (Y-11) antibody. In all cases, the immunoprecipitations were initially validated by screening membranes with the *anti*-HA peroxidase-conjugated (3F10POD) antibody to detect the HA-tagged hIP (Figure 10A, lower panel). In general, the resolution of the hIP from immunoprecipitations was poorer than that from direct protein analysis by SDS-PAGE, most likely due to high residual detergent in the former accounting for failure to resolve/detect all species of the hIP in the immunoprecipitates.

The *anti*-ubiquitin (P4D1) antibody revealed a diffuse ladder or smear of bands ranging from ~72 - 220 kDa in vehicle-treated HEK.hIP cells (Figure 10A, lane 5). Ubiquitinated forms of the hIP were also detected following stimulation of HEK.hIP cells with cicaprost (Figure 10A, lane 6), albeit at slightly reduced levels than in vehicle-treated cells (Figure 10A, compare lanes 5 & 6). The intensity of ubiquitination of the hIP was greatly increased when HEK.hIP cells were pretreated with MG132 either in the absence or presence of cicaprost (Figure 10A, lanes 7 & 8, respectively). As a control, non-transfected HEK 293 cells were treated in a similar manner to the HEK.hIP cells; however, no ubiquitinated species were detected in these cells (Figure 10A, lanes 1 - 4). Collectively, these data suggest that the hIP is subject to ubiquitination.

The immunoblot in Figure 10A is representative of the pattern detected when cells were lysed under non-denaturing conditions prior to immunoprecipitation. However, because a range of proteins can associate with the hIP, especially following its phosphorylation and internalisation, it was sought to establish whether the ubiquitin observed was directly associated with the hIP. To this end, HEK.hIP cells were lysed under denaturing conditions prior to immunoprecipitation. No differences in the patterns observed in Figure 10A, lanes 5 - 8, where the cells were lysed under non-denaturing conditions, were detected when HEK.hIP cells were lysed under denaturing conditions (data not shown). These data suggest that the ubiquitination was due to modification of the hIP itself rather than due to modification of protein(s) that actually bind or associate with the hIP.

The pattern of ubiquitination detected in the latter studies was characteristic of the hIP being subject to polyubiquitination rather than monoubiquitination. To verify this, HEK.hIP cells were treated with vehicle (0.001% DMSO) or MG132 and the HA-tagged hIP was immunoprecipitated using an *anti*-HA (Y-11) antibody followed by immunoblotting with an *anti*-ubiquitin (FK1) antibody, which recognises polyubiquitinated proteins only. Low levels of polyubiquitinated species were detected in HEK.hIP cells in the absence of proteasomal inhibition (Figure 10B, lane 3), but the levels increased dramatically when cells were treated with MG132 (Figure 10B, lane 4). As a control, no polyubiquitinated species were detected in vehicle-treated HEK 293 cells (Figure 10B, lane 1), while extremely low levels of polyubiquitinated species were detected in MG132-treated non-transfected HEK 293 cells (Figure 10B, lane 2). Therefore, these data suggest that the hIP was polyubiquitinated rather than monoubiquitinated or multi-monoubiquitinated.

**Figure 11:**
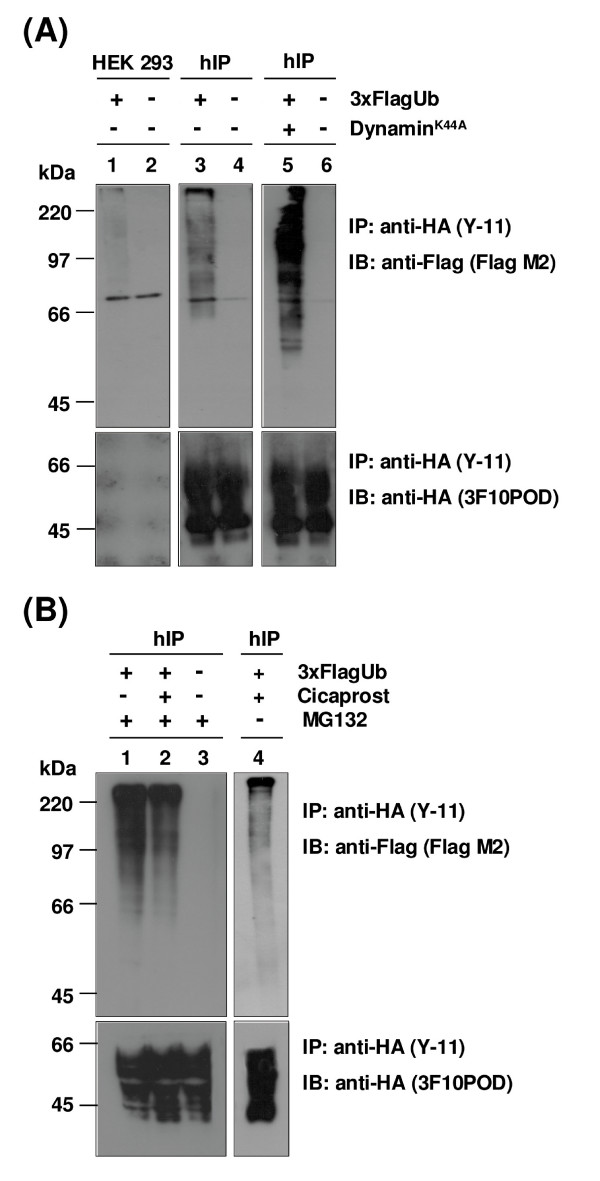
**Ubiquitination of the hIP**. **A**, HEK 293 (lanes 1 & 2) and HEK.hIP (hIP, lanes 3-6) cells were transiently transfected with pCMV5:3xFlag Ubiquitin (3xFlagUb), pcDNA3:HADynamin^K44A ^(Dynamin^K44A^) or, as controls, with the empty vectors pCMV5 or pcDNA3. **B**, HEK.hIP (hIP) cells were transiently transfected with pCMV5:3xFlag Ubiquitin (3xFlagUb) or, as control, with pCMV5 empty vector. Some 44 hr later, cells were treated with MG132 (10 μM) for 4 hr (lanes 1 & 3). Additionally, cells were pretreated with MG132 (10 μM) for 30 min prior to incubation with cicaprost (1 μM) for 4 hr (lane 2). As a control, cells were treated with cicaprost (1 μM) for 4 hr in the absence of MG132 (lane 4). **A **&**B**, thereafter, cells were harvested, lysed in Radio-immunoprecipitation (RIP) Buffer and HA-tagged hIPs were immunoprecipitated (IP) with the *anti*-HA antibody (IP: *anti*-HA (Y-11)). Immunoprecipitates were resolved by SDS-PAGE, followed by electroblotting to PVDF membrane. **A **&**B**, membranes were initially immunoblotted (IB) with the *anti*-HA peroxidase-conjugated antibody (IB: *anti*-HA (3F10POD), lower panels) to detect the HA-tagged hIP and were rescreened with the *anti*-Flag monoclonal antibody (IB: *anti*-Flag (Flag M2), upper panel) to detect Flag-tagged ubiquitinated species. The positions of the molecular size markers (kDa) are indicated to the left of **A **&**B**. Data are representative of three independent experiments.

In further efforts to validate ubiquitination of the hIP, HEK.hIP cells were cotransfected with or without a plasmid encoding 3xFlag-tagged Ubiquitin (3xFlagUb). Following cell lysis, the HA-tagged hIP was immunoprecipitated using the *anti*-HA (Y-11) antibody and, in all cases, immunoprecipitations were validated by immunoblotting for the HA-tagged hIP (Figure 11A, lower panels). Blots were then rescreened using a monoclonal *anti*-Flag M2 antibody specific for the Flag epitope. No ubiquitinated species were detected in HEK.hIP cells in the absence of cotransfection with 3xFlagUb (Figure 11A, upper panels, lane 4), thereby indicating the specificity of the *anti*-Flag antibody. The characteristic smear of bands representing polyubiquitinated species was evident when HEK.hIP cells were transfected with 3xFlagUb (Figure 11A, upper panels, lane 3). Very low levels of ubiquitinated species were detected when HEK 293 cells were transfected with 3xFlagUb (Figure 11A, upper panels, lane 1); however, these were significantly lower than the levels seen in HEK.hIP cells (Figure 11A, upper panels, compare lanes 3 & 1).

Ubiquitination of transmembrane proteins located at the ER membrane or the plasma membrane occurs on the cytosolic or intracellular domains. It has been previously reported that the hIP is internalised in a manner dependent, at least in part, on the GTPase dynamin [25]. Therefore, inhibition of hIP internalisation through co-expression of a dominant negative dynamin^K44A ^enhances the level of hIP retained at the plasma membrane, thereby increasing the possibility of detecting ubiquitinated hIP, as has been reported for other ubiquitinated GPCRs [37,52]. Hence, HEK.hIP cells were cotransfected with 3xFlagUb and dynamin^K44A ^and the HA-tagged hIP was immunoprecipitated using the *anti*-HA (Y-11) antibody prior to immunoblotting versus the *anti*-Flag antibody. Indeed, co-expression of dynamin^K44A ^dramatically increased the level of ubiquitinated species of the hIP detected compared to levels in cells transfected with 3xFlagUb alone (Figure 11A, upper panel, compare lane 5 to lane 3). As a control, no Flag-tagged ubiquitinated species were detected in the absence of cotransfection with 3xFlagUb or dynamin^K44A ^(Figure 11A, upper panels, lane 6). Therefore, these data suggest that the mature hIP may be polyubiquitinated.

Finally, HEK.hIP cells were then cotransfected with 3xFlagUb and treated with MG132 in the presence or absence of cicaprost prior to immunoprecipitation of the HA-tagged hIP. The immunoprecipitations were validated by initially immunoblotting using the *anti*-HA 3F10POD antibody (Figure 11B, lower panel). High levels of Flag-tagged ubiquitinated species were observed in the absence of agonist (Figure 11B, upper panel, lane 1), while ubiquitinated species were also detected when cells were pretreated with cicaprost (Figure 11B, upper panel, lane 2). As a control, HEK.hIP cells were cotransfected with 3xFlagUb and treated with cicaprost in the absence of MG132 (see lane 4 in the additional panel to the right of Figure 11B). While ubiquitinated species were detected in the presence of cicaprost alone, the levels were slightly lower than those seen when cells were treated with both cicaprost and MG132 (Figure 11B, compare lane 2 to lane 4). These data indicate that inhibition of the proteasomal degradation pathway with MG132 significantly increases the level of ubiquitinated species detected in HEK.hIP cells cotransfected with 3xFlagUb (compare Figure 11A, lane 3 to Figure 11B, lane 1).

**Figure 12:**
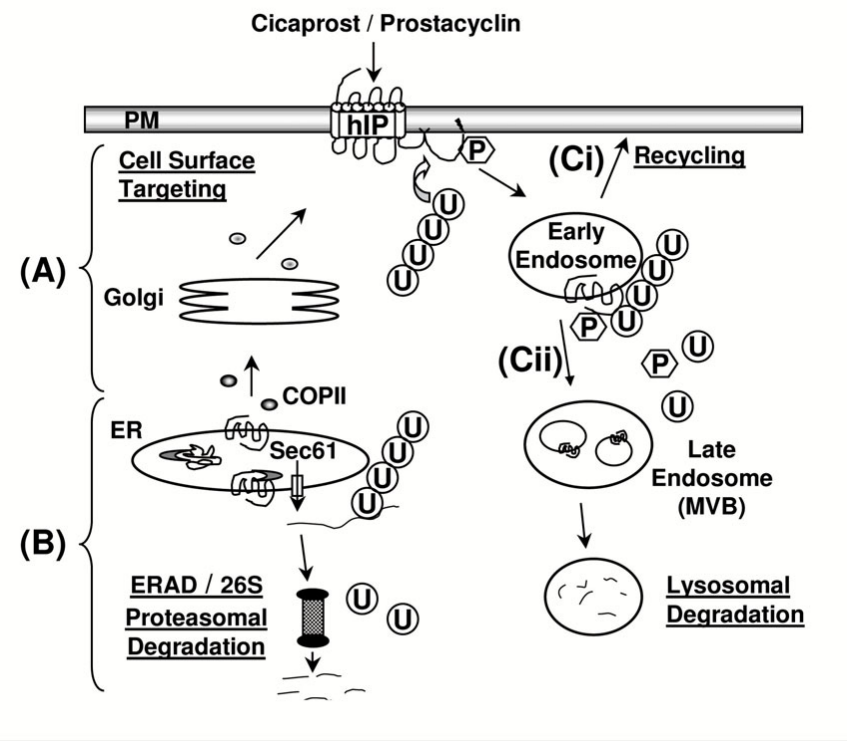
**Proposed model of the mechanisms mediating degradation of the hIP**. **(A)**, synthesis of the hIP begins in the rough ER. The hIP is then subject to co-and post-translational modifications, such as *N*-linked glycosylation and disulphide bond formation which, along with interaction with essential molecular chaperones, assist it to fold into its native conformation. Correctly folded receptors transit via ER exit sites and are subsequently transported in coatomer protein (COP)II vesicles to the Golgi complex. Further post-translational modification of the hIP may occur in the Golgi complex before the fully mature hIP is targeted to the plasma membrane. P indicates that the hIP may be phosphorylated. **(B)**, terminally misfolded hIPs may, however, be retrotranslocated to the cytosol, possibly through the Sec61 channel. Once the polypeptide chain is accessible in the cytosol, the hIP is polyubiquitinated and, following release from the ER membrane, is degraded by the 26S proteasomes through the ER-associated degradation (ERAD) system. **(C)**, following agonist-induced activation, the mature hIP is internalised into early Rab5a positive-endosomes. From there, it may be (i) recycled to the plasma membrane or (ii) polyubiquitinated which targets it to the late endosomes/multi-vesicular bodies (MVBs) and, thereafter, to the lysosomes for degradation.

Taken together, as presented in Figure 12, our data suggests a model in which the hIP may be degraded by two alternative pathways. In the first pathway (B), it is proposed that newly synthesised but misfolded species of the hIP may be retrotranslocated from the ER to the cytosol, most likely through the Sec61 channel [53]. Such retrotranslocated species may then be polyubiquitinated and targeted to the 26S proteasomes through the ERAD system [40,49]. The proteasome inhibitors MG132 or epoxomicin, but not the calpain inhibitor PD150606, leads to the accumulation of 4 predominant immature forms of the hIP representing the core glycosylated, non-farnesylated; core glycosylated, farnesylated; non-glycosylated, non-farnesylated; and non-glycosylated, farnesylated species, respectively. In general, it is thought that approximately 40% of newly synthesised wild-type proteins fail to mature correctly in the ER and, thus, are removed by ERAD [54,55]. Moreover, polyubiquitination and proteasomal degradation of several misfolded GPCRs has been established including the δ-opioid receptor [41], rhodopsin [56,57], the thyrotropin-releasing hormone receptor [58] and the calcium-sensing receptor [59]. The critical importance of ERAD is demonstrated by the fact that if the quality control system in the ER breaks down it can lead to the accumulation of proteins into aggregates in cells, resulting in such pathophysiologic conditions as Alzheimer's disease [60]. In addition, herein, while the proteasomes do not appear to be required for degradation of the mature hIP, viable proteasomes were required to maintain the level of functional receptor as indicated from the radioligand-binding studies and assessment of agonist-induced [Ca^2+^]_*i *_mobilization. A possible explanation for this reduced functional expression of the hIP evident in the presence of proteasomal inhibition may be that misfolded or misassembled receptors that accumulate in the presence of MG132, and which would normally be taken out/degraded by ERAD, escape the ERAD system and may thus be trafficked to the plasma membrane leading to accumulation of non-functional receptors.

In the second pathway of degradation (Figure 12C), it is proposed that the mature hIP may be subject to basal or agonist-induced degradation in the lysosomes whereby the inhibitors chloroquine and E64 specifically impaired turnover of the 46-66 kDa species of the hIP. However, data obtained herein, particularly following co-expression of 3xFlagUb and dynamin^K44A ^and in response to cicaprost, provides evidence that some of the mature hIP at least may also be polyubiquitinated and targeted to the lysosomes for degradation (Figure 11). Whilst MG132 did not appear to significantly affect the overall level of the mature hIP (46-66 kDa), a number of independent approaches herein confirmed that it may also be polyubiquitinated. Agonist-dependent ubiquitination of a number of mammalian GPCRs has been established, including the chemokine CXCR4 receptor [37,61], the V2 vasopressin receptor [33], the protease-activated receptor 2 [34] and the neurokinin 1 receptor [62]. Moreover, in each case, inhibition of ubiquitination of these GPCRs did not affect their internalisation into early endosomes, but does prevent their subsequent lysosomal trafficking and degradation. Whether ubiquitination of the mature hIP is required for its internalisation from the plasma membrane or whether ubiquitination of the mature hIP occurs independently of its internalisation but is required solely for its targeting to the lysosomal degradative system remains to be determined. It is generally accepted that ubiquitin is attached to substrates via covalent linkage of the C-terminus of ubiquitin to the ε-amino group of Lys residues of target proteins [29]. The hIP contains one Lys in intracellular loop III (Lys^218^) and four Lys residues (Lys^297^, Lys^304^, Lys^342 ^and Lys^376^) in its C-tail that are potential targets for ubiquitination. Studies are currently underway to identify which Lys residue(s) of the hIP are actually ubiquitinated.

As in many of the other aforementioned studies investigating basal and agonist-induced turnover and ubiquitination of GPCRs, largely owing to the levels of endogenous receptor expression the studies presented herein for the hIP are also exclusively based on a heterologous expression system. Moreover, other experimental approaches, such as mass spectrometry, may more accurately or conclusively confirm the identity of the various species of the hIP identified herein under the various conditions. Hence, it is indeed plausible that some of the observed effects herein, such as those in the presence of MG132, may be accentuated due to impairment or inability of the processing systems to deal with the level of hIP expression in the recombinant HEK.hIP cell line. Not withstanding this and the fact that the hIP is expressed at significantly lower levels physiologically within the vasculature, such as in platelets or VSMC [23,63], none the less the data presented substantially advances knowledge of the mechanisms of processing and maturation of the hIP, a complex if not somewhat unique GPCR that undergoes multiple post-translational modifications including phosphorylation, isoprenylation, palmitoylation, *N*-glycosylation, in addition to ubiquitination as determined herein. Knowledge of the roles of ubiquitination of the hIP significantly enhances understanding of the mechanisms regulating the function of the wild type hIP but may also, at least in part, provide a possible explanation as to why numerous nonsynonymous mutations, leading to dysfunctional hIP, have been increasingly recognized and are associated with a range of vascular disorders including venous thrombosis and intimal hyperplasia [10]. Moreover, knowledge of the factors determining the maturation and processing of the hIP may provide a rationale for the identification of potential therapeutic approaches to rescue hIP expression where normal physiologic function is altered.

## Conclusion

It was established that the hIP is subject to low-level basal degradation but degradation is enhanced in response to agonist. While the mature hIP may be polyubiquitinated and targeted to the lysosomal pathway, the misfolded immature species may be polyubiquitinated, retrotranslocated from the endoplasmic reticulum and degraded by the 26S proteasomes through the ERAD system. These data substantially advance knowledge of the factors regulating the processing and maturation of the hIP, a complex GPCR subject to multiple post-translational modifications including N-glycosylation, phosphorylation, isoprenylation, palmitoylation, in addition to polyubiquitination as determined herein.

## Methods

### Materials

Cicaprost and SCH66336 were gifts from Bayer Schering Pharma AG and Schering-Plough, Inc., respectively. [^3^H]iloprost (15.3 Ci/mmol) was from Amersham Biosciences. Cycloheximide (CHX), epoxomicin, E-64, PD150606 and Fura2/AM were from Calbiochem. Peptide-N-glycosidase F (PNGase F) and recombinant endoglycosidase H (Endo H) were obtained from New England Biolabs. The chemiluminescence western blotting kit was from Roche. Effectene^® ^transfection reagent was from Qiagen. Tunicamycin, chloroquine (CLQ), MG132, lactacystin, N-ethyl maleimide (NEM), protein A-sepharose and mouse monoclonal *anti*-FLAG M2 antibody (F 3165) were obtained from Sigma-Aldrich. Hemagglutinin (HA)-probe (Y-11, sc-805) rabbit polyclonal antibody, mouse monoclonal *anti*-ubiquitin (P4D1, sc-8017) antibody and horse radish peroxidase (HRP)-conjugated goat *anti*-rabbit IgG, goat *anti*-mouse IgG and goat *anti*-mouse IgM secondary antibodies were purchased from Santa Cruz Biotechnology. Mouse monoclonal *anti*-polyubiquitin (FK1, PW 8805) antibody was from Biomol International. Mouse monoclonal *anti*-HDJ-2 antibody was from Neomarkers. The oligonucleotides used in this study were synthesised by Sigma-Genosys.

### Plasmids

The plasmid pHM6:hIP has been previously described [42]. The plasmids pCMV5:3xFlag Ubiquitin (3xFlagUb) and pcDNA3:HA:Dynamin^K44A ^were kind gifts from Dr J. Benovic, Thomas Jefferson University, PA, U.S.A.

### Cell Culture and Stable Cell Lines

Human embryonic kidney (HEK) 293 cells, obtained from the American Type Culture Collection, were cultured in minimum essential medium (MEM), supplemented with 10% foetal bovine serum (FBS). The HEK.hIP cell line stably overexpressing a HA-tagged form of the hIP has been previously described [42].

### Cell Treatment, SDS-PAGE and Western Blotting

To investigate hIP turnover, HEK.hIP cells were seeded at 2 × 10^5 ^cells/well in 6-well plates and grown for 48 - 72 hr. Thereafter, cells were treated with a range of compounds, generally for 0 - 12 hr, as outlined in the *Results *section and in the respective figure legends. Cells were harvested in 1 × PBS, pH 7.4 containing protease inhibitors (0.5 mM phenylmethylsulfonyl fluoride (PMSF); 2.0 mM 1, 10 phenanthroline; 10 μg/ml aprotinin; 1 mM benzamidine hydrochloride; 1 μg/ml leupeptin). In all cases, protein concentration was assayed using the Bradford assay [64]. Generally, 50 μg total cellular protein was resuspended in SDS-sample Buffer (10% β-mercaptoethanol (v/v); 2% SDS (w/v); 30% glycerol (v/v); 50 mM Tris-HCl, pH 6.8; 0.025% bromophenol blue (w/v)). Samples were then boiled for 10 min prior to resolution by SDS-PAGE, on 10% polyacrylamide gels, followed by electroblotting onto PVDF membrane. Blots were screened versus the *anti*-HA (3F10; 1:1,000) antibody, followed by the secondary HRP-conjugated goat *anti*-rat IgG antibody (1:5,000), and HA-tagged proteins were visualised using the chemiluminescence detection system, as described by the supplier. Membranes were then stripped and reprobed using the *anti*-HDJ-2 antibody (1:4,000) to ensure equal protein loading. All blots illustrated in the figures are representative blots from 3 - 6 experiments (n = 3 - 6), where the exposures presented are those that best represent the experimental findings of all replicates. As relevant and deemed necessary, two exposures (short and long) of certain data is presented for clarity. To quantify changes in the levels of the mature hIP, regions of the *anti*-HA immunoblots corresponding to the 46-66 kDa region were captured using Adobe Photoshop (V6); band widths and intensities were quantified and represented as levels of HA-hIP expression under the various treatment conditions and expressed as a percentage of the level of HA-hIP expression under control/non-treated conditions.

### Cell Fractionation

HEK.hIP cells were seeded at 2 × 10^6 ^cells/100-mm dish and grown for 48 hr. Cells were then treated with MG132 (10 μM) or, as control, with vehicle (0.001% DMSO) for 12 hr prior to harvesting. An aliquot of total (T) cell protein was retained and the remaining cells were resuspended in Homogenisation Buffer (25 mM Tris/HCl, pH 7.5; 0.25 M sucrose; 10 mM MgCl_2_; 1 mM EDTA; 0.1 M PMSF), homogenised on ice for 60 sec and then centrifuged at 100,000 × *g *for 60 min at 4°C. The soluble, supernatant fraction (S_100_) was retained for analysis and the pellet fraction (P_100_), representing crude membranes, was washed in MES-KOH buffer (10 mM MES-KOH, pH 6.0; 10 mM MnCl_2_; 1 mM EDTA; 10 mM indomethacin) prior to resuspension in 10 mM Tris-Cl, 1 mM EDTA, pH 8.0. Aliquots of the total, P_100 _and S_100 _fractions (50 μg/lane) were then resolved by SDS-PAGE followed by immunoblot analysis, as previously outlined.

### Deglycosylation Reactions

HEK.hIP cells were seeded at 2 × 10^6^cells/100-mm dish and grown for 48 hr. Cells were then pretreated in the presence (+) or absence (-) of SCH66336 (5 nM, 24 hr) or MG132 (10 μM, 12 hr), as outlined in the respective figure legends. Cells were harvested and aliquots (50 μg) of total cell protein digested with PNGase F or Endo H, essentially according to the suppliers' instructions. Briefly, cellular protein was denatured in 1 × Glycoprotein Denaturing Buffer (0.5% SDS, 40 mM dithiothreitol) at 100°C for 10 min. Thereafter, for digestions involving PNGase F, 1 × G7 Reaction Buffer (50 mM sodium phosphate, pH 7.5) and 1% NP-40 were added prior to addition of PNGase F (500 units/50 μg protein). For digestions involving Endo H, 1 × G5 Reaction Buffer (50 mM sodium citrate, pH 5.5) was added prior to addition of 1 μl Endo H (200 units/50 μg protein). All reactions were incubated at 37°C for 16 hr and were terminated by addition of an equal volume of SDS-sample Buffer and prepared for SDS-PAGE and immunoblotting, as previously outlined herein.

### Investigation of Ubiqutination of the hIP

HEK.hIP cells or, as control, HEK 293 cells were seeded at 2 × 10^6 ^cells/100-mm dish and grown for 72 hr. Cells were treated with MG132 (10 μM), cicaprost (1 μM), MG132 (10 μM) for 30 min followed by stimulation with cicaprost (1 μM) or, as control, with vehicle (0.001% DMSO) for 4 hr, as outlined in the respective figure legends. Cells were then lysed in Radio-immunoprecipitation (RIP) Buffer (50 mM Tris-HCl, pH 8.0, 150 mM sodium chloride, 5 mM EDTA, 1% NP-40 (v/v), 0.5% sodium deoxycholate (w/v), 0.1% SDS (w/v)) supplemented with protease inhibitors (0.5 mM PMSF; 2.0 mM 1, 10 phenanthroline; 10 μg/ml aprotinin; 1 mM benzamidine hydrochloride; 1 μg/ml leupeptin). Immunoprecipitations were carried out in the presence of N-ethyl maleimide (NEM, 10 mM), an alkylating agent that inhibits the cellular deubiquitinating enzymes [65]. When performing immunoprecipitations under denaturing conditions, cells were lysed in RIP Buffer supplemented with 1.0% SDS. Following 15 min incubation on ice, cells were harvested and disrupted by sequentially passing through hypodermic needles of decreasing bore size (18-, 21-, 23- and 26-gauge). Samples were centrifuged at 55,000 × *g *for 30 min at 4°C and HA-tagged receptors from resulting supernatants (~600 μg) were immunoprecipitated with *anti*-HA antibody (Y-11, sc-805, 1:30 dilution) at 4°C for 1 hr. Protein A-Sepharose (50% slurry in PBS; 50 μl) was then added and samples were incubated at 4°C for a further 60 min. Immune complexes were collected by centrifugation at 12,000 × *g *at 4°C for 5 min. The supernatants were removed and the beads were washed by resuspension in 600 μl RIP Buffer and then incubated at 4°C for 10 min. Immune complexes were collected by centrifugation at 12,000 × *g *at 4°C for 5 min and this washing process was repeated a further three times. The last traces of RIP Buffer were removed and proteins were eluted from the beads with 30 μl of SDS-sample Buffer. Generally, samples were then boiled for 10 min prior to resolution by SDS-PAGE, on 8% polyacrylamide gels, followed by electroblotting onto PVDF membrane. Prior to immunoblotting, membranes were treated with Denaturation Solution (62.5 mM Tris-HCl, pH 6.7, 100 mM β-mercaptoethanol, 2% SDS) for 30 min at 60°C. While proteins subjected to SDS-PAGE are usually sufficiently denatured, PVDF membranes were treated with this solution to further denature the proteins prior to immunoblotting, thereby optimising conditions to enable the detection of ubiquitinated proteins [37]. Membranes were screened with *anti*-HA peroxidase-conjugated antibody (3F10POD, 1:500) to detect HA-tagged receptors. Subsequently, membranes were re-treated with Denaturation Solution to remove bound antibody and were reprobed with a mouse monoclonal *anti*-ubiquitin (mono- and polyubiquitination) primary antibody (P4D1, 1:5,000), followed by a secondary screening with a HRP-conjugated goat *anti*-mouse IgG antibody (1:10,000). Alternatively, membranes were reprobed with a mouse monoclonal *anti*-ubiquitin (polyubiquitin) primary antibody (FK1, 1:5,000), followed by a secondary screening with a HRP-conjugated goat *anti*-mouse IgM antibody (1:10,000). Membranes were screened using the chemiluminescence detection system, as described by the supplier (Roche).

Additionally, ubiquitination of the wild-type hIP was investigated following cotransfection of HEK.hIP cells with pCMV5:3xFlag Ubiquitin (3xFlagUb) and/or pcDNA3:HA:Dynamin^K44A^. Specifically, HA-tagged HEK.hIP or, as control, HEK 293 cells were seeded at a density of 2 × 10^6 ^cells/100-mm dish. After 48 hr, cells were transiently transfected with 1 μg total DNA, comprising 270 ng pAdVA [66] plus 730 ng of the plasmid encoding 3xFlagUb alone or 365 ng of 3xFlagUb plus 365 ng of pcDNA3:HA:Dynamin^K44A ^or, as control, with an equivalent amount of the respective empty vectors, pCMV5 or pcDNA3, essentially as outlined in the respective figure legends. Cells were then cultured for a further 48 hr. Where indicated, HEK.hIP cells were treated with MG132 (10 μM) for 4 hr or were pretreated with MG132 (10 μM) for 30 min prior to stimulation with cicaprost (1 μM) for 4 hr. Thereafter, cells were lysed in RIP buffer supplemented with protease inhibitors (0.5 mM PMSF; 2.0 mM 1, 10 phenanthroline; 10 μg/ml aprotinin; 1 mM benzamidine hydrochloride; 1 μg/ml leupeptin) and NEM (10 mM) under non-denaturing or denaturing conditions, as previously outlined. Supernatants were obtained and HA-tagged receptors were immunoprecipitated by incubation with *anti*-HA antibody (Y-11), as previously outlined. Immunoprecipitates were resuspended in SDS-sample Buffer, boiled for 10 min and resolved by SDS-PAGE, on 8% polyacrylamide gels, followed by electroblotting onto PVDF membrane. Prior to immunoblotting, membranes were treated with Denaturation Solution and were screened with *anti*-HA peroxidase-conjugated antibody (3F10POD, 1:500) to detect HA-tagged receptors. Subsequently, membranes were re-treated with Denaturation Solution and reprobed with *anti*-Flag M2 mouse monoclonal antibody (1:5,000), followed by incubation with a secondary HRP-conjugated goat *anti*-mouse IgG antibody (1:10,000). Membranes were screened using the chemiluminescence detection system, as described by the supplier.

### Radioligand-binding Assays

Radioligand-binding assays (RLBAs) of the hIP, over-expressed in HEK.hIP cells, were carried out on crude cell membranes (P_100_) using 4 nM [^3^H]iloprost essentially as previously described [67]. To assess the effect of MG132 on the expression levels, HEK.hIP cells were pretreated with MG132 (10 μM) for 3, 6, 9 or 12 hr prior to harvesting. As a control, HEK.hIP cells were incubated with the vehicle (0.001% DMSO) for the same times. Thereafter, cells were washed three times with 1 × PBS to remove all traces of MG132 and RLBAs were carried out as previously described [67].

### Measurement of Intracellular Ca^2+ ^Mobilisation

Measurement of agonist-induced intracellular Ca^2+ ^([Ca^2+^]_*i*_) mobilisation in Fura2/AM-preloaded cells was carried out essentially as previously described [68]. To assess the effect of MG132 on cicaprost-induced [Ca^2+^]_*i *_mobilisation, HEK.hIP cells were pretreated with MG132 (10 μM) for 3, 6 or 12 hr. As controls, HEK.hIP cells were left untreated or were pretreated with vehicle (0.001% DMSO) for 12 hr. Thereafter, Fura2/AM-preloaded cells were stimulated with the IP agonist cicaprost (1 μM). Data presented are representative of at least three independent experiments and were calculated as mean changes in intracellular Ca^2+ ^mobilised (Δ[Ca^2+^]_*i *_± S.E.M., nM; n = 4) as a function of time (s) following ligand stimulation.

## Data Analyses

Statistical analyses were carried out using the unpaired Student's *t*-test or, where relevant and specifically indicated in the text, using two-way ANOVA employing the GraphPad Prism package. *P*-values of = 0.05 were considered to indicate a statistically significant difference.

## Abbreviations

AC: adenylyl cyclase; C-tail: carboxyl-terminal tail; [Ca^2+^]_*i*_; intracellular calcium; CHX: cycloheximide; CLQ: chloroquine; Endo H: endoglycosidase H; ER: endoplasmic reticulum; ERAD: ER-associated degradation; FTI: farnesyl transferase inhibitor; GPCR: G protein-coupled receptor; HA: hemagglutinin; HEK: human embryonic kidney; hIP: human IP; IP: PGI_2 _receptor; MW: molecular weight; NEM: N-ethyl maleimide; PG: prostaglandin; PK: protein kinase; PL: phospholipase; PNGase F: peptide-N-glycosidase F; RIP: radio-immunoprecipitation; SMC: smooth muscle cell.

## Competing interests

The authors declare that they have no competing interests.

## Authors' contributions

PDD performed the experiments and drafted the manuscript. BTK designed the study and critically revised and evaluated the manuscript. All authors read and approved the final manuscript.
